# Development of the Japanese Core Outcome Measures Index (COMI): cross-cultural adaptation and psychometric validation

**DOI:** 10.1186/s12891-018-1986-x

**Published:** 2018-03-02

**Authors:** Ko Matsudaira, Hiroyuki Oka, Yasushi Oshima, Hirotaka Chikuda, Yuki Taniguchi, Yoshitaka Matsubayashi, Mika Kawaguchi, Emiko Sato, Haruka Murano, Thomas Laurent, Sakae Tanaka, Anne F. Mannion

**Affiliations:** 1 Department of Medical Research and Management for Musculoskeletal Pain, 22nd Century Medical and Research Center, Faculty of Medicine, The University of Tokyo, Tokyo, Bunkyo-ku Japan; 2 Department of Pain Medicine, Fukushima Medical University School of Medicine, Fukushima, Japan; 3 Department of Orthopaedic Surgery, The University of Tokyo, Tokyo, Bunkyo-ku Japan; 4 Department of Orthopedic Surgery, Gumma University Graduate School of Medicine, Maebashi, Japan; 5 Clinical Study Support, Inc., Nagoya, Japan; 6 Spine Center Division, Department of Teaching, Research and Development, Schulthess Klinik, Zürich, Switzerland

**Keywords:** Cross cultural adaptation, Psychometric validation, Core outcome measures index, Low back pain, Degenerative disorders of the lumbar spine

## Abstract

**Background:**

The patient-rated Core Outcome Measures Index (COMI) assesses the multidimensional impact of back problems on the sufferer. The brevity and comprehensibility of the tool make it practical for use in clinical and research settings. Although the COMI has been cross-culturally adapted in various languages worldwide, there is currently no Japanese version. The aim of this study was to develop a Japanese version of the COMI by: (1) performing a cross-cultural adaptation of the English version and (2) evaluating the psychometric properties of the Japanese version of the COMI in Japanese volunteers with chronic back problems.

**Methods:**

The English version of the COMI was cross-culturally adapted for the Japanese language using established guidelines. The pre-final version was pilot-tested in five Japanese-speaking patients with low back pain (LBP) and a history of spine surgery. The psychometric properties of the Japanese COMI were tested in a group of 1052 individuals with chronic LBP (LBP ≥3 months), aged 20–69 years, who were recruited through a web-based survey. The psychometric properties that were evaluated included convergent and known-group validity, using the following reference questionnaires: EuroQol 5 Dimension, Roland Morris Disability Questionnaire, Short Form 8™ Health Survey, and the Keele STarT Back Screening Tool.

**Results:**

The pre-final version of the cross-culturally adapted Japanese COMI was completed without any major problems of understanding or acceptability. For the evaluation of its psychometric properties, tests for convergent validity showed moderate correlations between COMI items and the respective reference questionnaires for symptom-specific well-being [− 0.33–−0.48] and disability domains [0.48] and strong correlations (> 0.5) for the other domains and the COMI summary score. The analysis of known-group validity showed a linear trend for the COMI score in relation to prognostic risk (*P* < 0.001).

**Conclusions:**

The Japanese COMI retained conceptual equivalence to the original using comprehensible and acceptable Japanese expressions. We developed a Japanese version of the COMI that displayed qualities that support its convergent and known-group validity. The availability of a Japanese version of the COMI should allow for improved documentation of the care provided to patients with back problems.

**Electronic supplementary material:**

The online version of this article (10.1186/s12891-018-1986-x) contains supplementary material, which is available to authorized users.

## Background

Low back pain (LBP) is a common, disabling health problem. Although its prognosis is mostly benign [1], 2–7% of patients develop chronic LBP [2]. Since chronic LBP affects patients’ lives beyond physical pain and disability, assessments that encompass multidimensional self-reported outcomes are required for documenting the impact of LBP and the response to treatment.

The Core Outcome Measures Index (COMI) was developed to evaluate the multidimensional impact of LBP. It was based on a set of single questions (concerned with pain symptoms, function, symptom-specific well-being, and disability) that had been recommended for use by an expert group [3]. These items, and an additional question on general quality of life, were subsequently put together and validated as an index [4]. With established reliability, validity, and responsiveness [4–7], the brief but comprehensive coverage of the COMI alleviates response burden on patients, rendering the COMI a practical tool in clinical and research settings.

Since its initial development, the availability and use of the COMI has expanded: it has been cross-culturally adapted for an array of different languages, and these language versions have displayed good psychometric properties [4–6, 8–11]. It has also been modified for use in patients with neck problems [7, 12]). To date, however, no Japanese version has been developed. In order to apply this parsimonious and practical tool in Japanese clinical settings, a need was seen for the COMI to be cross-culturally adapted for use in Japanese patients. The availability of a Japanese version of the COMI would promote the wider use of the questionnaire and allow for improved documentation of care in Japanese patients with back problems.

The aim of this study was to develop a Japanese version of the COMI by: (1) performing a cross-cultural adaptation of the English version and (2) evaluating the psychometric properties of the Japanese version of the COMI in volunteers with chronic back problems, resident in Japan.

## Methods

The English version of the COMI was cross-culturally adapted for the Japanese language, in accordance with previously published guidelines [13, 14], and its psychometric properties were evaluated in data collected in a cross-sectional survey. Ethical approval was obtained from the ethical committee of The University of Tokyo [Approval number: 10665-(1)]. All participants in the survey gave their consent electronically and were compensated with vouchers (e.g., shopping points). No personally identifiable information was collected.

### COMI

COMI comprises seven items: back pain, leg/buttock pain, function, symptom-specific well-being, general quality of life, social disability, and work disability. All items refer to the last week, except for the two disability items (past 4 weeks). Back and leg/buttock pain are rated on separate 10-point graphic rating scales; the other items are responded to using a 5-point scale. A higher score indicates a worse status.

Scores are calculated for each domain and for the summary score [15]. For the latter, the higher score of the back or leg/buttock pain is first taken as the pain domain score. Then, the other item scores are converted from their 5-point scales into a 0 to 10-point range using increments of 2.5 (0, 2.5, 5.0, 7.5, 10.0). Social and work disability scores are averaged to form one disability domain score. Averaging the five domain scores (now each scored 0–10) — pain, function, symptom-specific well-being, general quality of life, and disability — yields a summary score ranging from 0 to10 (best to worst health status) [4, 6].

The Japanese COMI questions were supplemented with another question to identify the predominant problem [16], using an item from the Spine Tango patient self-assessment form [17]. This independent item is not included in the COMI scoring [16]. The item enquires as to which problem is the most troublesome (back pain, leg pain, sensory disturbances, or other).

### Cross-cultural adaptation

#### Translation and synthesis

Two native Japanese speakers (an expert in the measured concept and clinical contents of the questionnaire and a layperson not familiar with the concept) independently translated the original version into Japanese. Their different profiles and backgrounds were expected to enhance conceptual and semantic equivalence. The two translations were compared with each other and with the original. After any discrepancies were resolved by discussion and consensus, the two translations were synthesized into one Japanese consensus version.

#### Back-translation

Two native English speakers blinded to the original English version and not familiar with the concepts independently back-translated the Japanese consensus version into English.

#### Expert committee

Two forward-translators, one methodologist (a researcher with experience in cross-cultural adaptations), and one clinician constituted an expert committee to produce a pre-final version of the Japanese COMI by consolidating all the translated versions in close contact with the developer of the COMI and the back translators. The committee members reviewed and discussed all the translations to assure semantic and conceptual equivalence between the original and translated versions. All the processes and rationales involved prior to reaching a consensus were documented in written form.

#### Pilot-test

Five Japanese-speaking patients with LBP and a history of spine surgery completed the pre-final version. After completion, the patients were debriefed regarding their general comments on the instrument and their understanding of the questions, to confirm comprehensibility and conceptual equivalence. Debriefing results were reviewed and the findings were used in producing the final version of the Japanese COMI.

### Psychometric validation

#### Participants

**Fig. 1:**
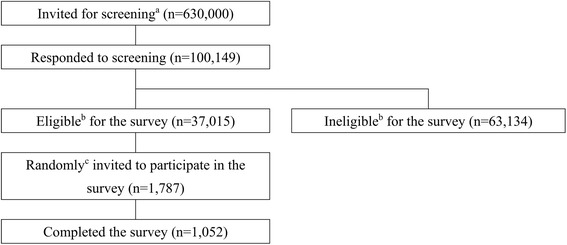
Participant recruitment flow for the Japanese COMI validation study. *a* Of the whole registrants to the Internet research company, 630,000 in the eligible age range (20–69 years) were randomly invited to participate in the screening survey. *b* Screening respondents were considered eligible for the survey if they had chronic LBP (LBP lasting for ≥3 months) with severity graded as follows: I, no interference with everyday activities; II, interference with everyday activities but no absence from social activities including work, housework, and school; or III, interference with social activities, leading to absence from social activities [18]; but without LBP caused by cancer, inflammation, aneurysm, urolithiasis, or fracture. *c* Eligible participants were randomly selected based on computer-generated randomization sequences, stratified by sex and LBP severity, in order to obtain an equal number of males and females in each severity group. A total of 1787 eligible participants who were registered at the time of our survey were invited to take part in the study; 13 patients who withdrew registration to the panel after the screening were not invited

Evaluation of the psychometric properties of the Japanese COMI was carried out in individuals with chronic LBP, aged 20–69 years. We recruited participants through a web-based survey outsourced to the Internet research company, IDEA PROGGET Co., Ltd. (Tokyo, Japan). Any individual residing in Japan who is aged ≥15 years and is interested in online surveys can register themselves with the research company and can freely choose whether they wish to participate in a given survey, based on the invitation emails distributed by the research company. Figure 1 depicts the recruitment flow of the participants; 630,000 in the eligible age range were randomly selected from the registered individuals and invited to participate in the initial screening survey. Those individuals interested in the survey (*n* = 100,149) were screened for age and the presence of chronic LBP (defined as LBP lasting for ≥3 months) with severity graded as follows: I, no interference with everyday activities; II, interference with everyday activities but no absence from social activities including work, housework, and school; or III, interference with social activities, leading to absence from social activities [18]. Patients with LBP caused by cancer, inflammation, aneurysm, urolithiasis, or fracture were excluded. The screening yielded 37,015 participants satisfying the admission criteria.

After the screening, eligible participants were randomly selected based on computer-generated randomization sequences, stratified by sex and LBP severity, in order to obtain an equal number of males and females in each severity group. A total of 1787 eligible participants who were registered at the time of our survey were invited to take part in the study; 13 patients who withdrew registration to the panel after the screening were not invited. Those who were interested in the invitation responded to the questionnaire battery of their own free will.

#### Questionnaire battery

The questionnaire battery included questions regarding sociodemographic and clinical characteristics, the Japanese COMI, the EuroQol 5 Dimension (EQ-5D) [19], the Roland Morris Disability Questionnaire (RDQ) [20], the Short Form 8™ Health Survey (SF-8) [21], and the Keele STarT Back Screening Tool (STarT) [22] [Table 1].

**Table 1:** Reference questionnaires

Questionnaire	Content	Scale	Item number	Score range	COMI domains expected to correlate strongly
EQ-5D [18]	General health status				Summary, QOL
The descriptive system	Mobility, self-care, usual activities, pain/discomfort, and anxiety/depression	3-point Likert	5	−0.111–1.000^a^	
EQ-VAS		VAS	1	0–100^b^	
RDQ [19]	Physical disability due to low back pain	Dichotomous (yes or no)	24	0–24^c^	Summary, Function, Disability
SF-8 [20]	General health-related quality of life	5- or 6-point Likert	8		
PCS				5.32–70.69^d^	Summary, Pain, Function, Symptom-specific well-being, QOL
MCS				10.11–74.51^d^	Symptom-specific well-being, QOL
STarT [21]	Potentially modifiable prognostic indicators Five questions related to the psychological factors constitute the sub-score	5-point Likert and Dichotomous (agree or disagree)	9	0–9^e^	Summary, Pain, Function, Disability

^a^Calculated using the value set for the Japanese population [34]. The score of 1 denotes “full health” and 0 “death”

^b^The score of 0 indicates worst imaginable health state and 100 best possible health state

^c^A higher score indicates greater disability

^d^Calculated based on a norm-based scoring method given in the instrument guidelines [21]. A higher score indicates better health

^e^A total score of 0–3 indicates low prognostic risk; a total score of ≥4 with sub score of ≤3, medium prognostic risk; sub score of ≥4, high prognostic risk

EQ-5D: the EuroQol 5 Dimension; VAS: visual analogue scale; RDQ: Roland Morris Disability Questionnaire; SF-8: Short Form 8™ Health Survey; PCS: physical component summary; MCS: mental component summary; STarT: the Keele STarT Back Screening Tool

### Statistical analysis

The sociodemographic and clinical characteristics of the participants were summarized descriptively. To evaluate the ability of the Japanese COMI to capture the full range of the impact of LBP, we assessed floor and ceiling effects (percentage of individuals reporting worst and best status, respectively) for each COMI item and domain, for the COMI summary score, and also for the other questionnaires. Floor effects were considered present if > 15% of the participants achieved the worst status, and ceiling effects were considered present if > 15% of the participants achieved the best status [23].

The validity of the Japanese COMI was evaluated in terms of convergent validity and known-group validity. For convergent validity, the degree of correlation between each COMI domain or the COMI summary score with the reference questionnaire measuring the same or similar traits was measured using Spearman rank correlation coefficients. We considered a correlation coefficient of 0.1 as weak, 0.3, moderate, and 0.5, strong [24]. The correlation between the COMI and the corresponding reference questionnaires was expected to be strong [Table 1].

Known-group validity was evaluated by examining whether the COMI scores differed among the STarT prognostic risk groups. Participants were stratified into three risk groups based on the total score of the 9 questions and on the sub-score for the 5 psychological questions of the STarT: low risk (total score of 0–3), medium risk (total score of ≥4 with sub-score of ≤3), and high risk (sub score of ≥4) groups [22]. We used the Jonckheere-Terpstra test [25, 26] to test for a trend in the COMI summary score in association with the prognostic risk level. The non-parametric Jonckheere-Terpstra test evaluates the difference between the scores for a continuous variable among defined groups, taking the ordering of the groups (prognostic risk levels, in this study) into account.

Although not typically considered appropriate for multidimensional indexes [27], for the purposes of comparison with other language versions we assessed internal consistency of the Japanese COMI using the standardized Cronbach’s alpha, whereby coefficients above 0.7 are usually considered acceptable [28].

The Jonckheere-Terpstra test was one-sided and the other tests were two-sided. The significance level was set at 0.05. Data were analyzed using SAS software version 9.4 (SAS Institute, Inc., Cary, NC, USA).

## Results

### Cross-cultural adaptation

#### Translation

The following few items required modification in the forward translations: “pins and needles” (in the questionnaire instruction and “location of the main problem” item), “recreational activities” (social disability), “moderately” (response option of function), and “none” (response option of social and work disability). The concepts of “pins and needles” and “recreational activities” do not fully correspond to any concise Japanese expressions. For “moderately” and “none”, the literal forward-translations to reflect the equivalent order and intervals between the original response options were considered to make the translations unnatural to the respondents. We sought expressions that would retain the original concepts and at the same time be familiar to Japanese speakers. The former two were back-translated as follows: “pins and needles” as “prickling sensation”, and “recreational activities” as “engaging in hobbies, and recreation” or “vocational activities, or amusement activities”. For each of the response options (“moderately” and “none”), we left two tentative translations (back-translations: “moderately” and “somewhat” for “moderately”; and “none” and “0 days” for “none”), with the aim of selecting the most natural expression based on the pilot-test results (see below).

#### Pilot-test

All five Japanese LBP patients answered the pre-final version of the Japanese COMI without major problems in relation to comprehensibility and acceptability. Of the tentative options for “moderately” and “none”, we adopted expressions that back-translated as “moderately” and “none”, respectively, for the final version, based on the participants’ preferences and the conceptual equivalence to the original [Additional file 1].

### Psychometric validation

A total of 1052 participants completed the questionnaires (Fig. 1). Completion of the web survey required answers to all questions and thus there were no missing data. The mean (standard deviation [SD]) age of the participants was 48.3 (12.6) years [Table 2]; 63.5% were male and 67.9% had non-specific LBP. LBP severity was evenly distributed among the three grades, I to III (about 33% each), as intended. In total, 79.4% individuals had had LBP for more than 18 months, but 60.8% had not taken any sick leave and 75.2% were not currently receiving any treatment for LBP.

**Table 2:** Demographic and clinical characteristics of the participants (*n* = 1052)

Characteristics	n (%)
Sex	
Male	668 (63.5)
Age, year (mean ± SD)	48.3 ± 12.6
LBP before this episode	
Yes	869 (82.7)
Duration of current episode	
≥3–< 6 months	62 (5.9)
≥6–< 12 months	79 (7.5)
≥12–< 18 months	76 (7.2)
≥18 months	835 (79.4)
Severity of LBP	
Grade I^a^	351 (33.4)
Grade II^b^	353 (33.6)
Grade III^c^	348 (33.1)
Normal work	
Regular employee	405 (38.5)
Contract employee	171 (16.3)
Temporary employee	27 (2.6)
Business owner	76 (7.2)
Helping family business	8 (0.8)
Home worker	22 (2.1)
Student	3 (0.3)
Homemaker	136 (12.9)
Unemployed	178 (16.9)
Other	26 (2.5)
Length of current sick leave	
Not on sick leave	640 (60.8)
< 1 month	289 (27.5)
≥1–< 3 months	42 (4.0)
≥3–< 6 months	20 (1.9)
≥6–< 12 months	12 (1.1)
≥12–< 18 months	5 (0.5)
≥18 months	44 (4.2)
Educational level	
Junior High school	31 (2.9)
Secondary education	545 (51.8)
University education	427 (40.6)
Higher degree	46 (4.4)
Others	3 (0.3)
Type of work	
Sedentary	477 (45.3)
Physical	198 (18.8)
Others	377 (35.8)
Current treatment for LBP	
Yes	261 (24.8)
Radiation of current pain	
To the buttock or thigh but not to the knee	182 (17.3)
To the buttock, thigh, shin and feet	156 (14.8)
No	714 (67.9)
Non-specific/Specific LBP	
Non-specific	714 (67.9)
Disc herniation, spinal stenosis or both	121 (11.5)
Other cause of radiation of pain	217 (20.6)
Pain location (COMI)	
Low back pain and back pain	499 (47.4)
Leg/buttock pain	133 (12.6)
Sensory disturbances (back, leg or buttocks)	163 (15.5)
Other	257 (24.4)

Data are presented as n (%), unless otherwise specified

There were no missing data since the survey completion required answers to all questions

^a^No interference with everyday activities

^b^Interference with everyday activities but not with social activities including work, housework, and school

^c^Interference with social activities including work, housework, and school

SD: standard deviation; LBP: low back pain; COMI: core outcome measure index

#### Floor and ceiling effects

Table 3 shows the floor effects (worst status) and ceiling effects (best status) for the COMI and the reference questionnaires. The social and work disability items showed particularly high percentages for ceiling effects (72.5% and 82.9%, respectively).

**Table 3:** Scores and distribution of the Japanese COMI and reference questionnaires

Scale/Domain	mean (SD)	Median	Range Min–Max	Floor effect (worst status) (%)	Ceiling effect (best status) (%)
COMI^a^					
Back Pain	3.7 (2.6)	3.0	0–10	1.5	12.4
Leg Pain	2.6 (2.7)	2.0	0–10	1.1	33.3
Pain	4.0 (2.7)	4.0	0–10	1.8	10.7
Back Function	2.5 (2.5)	2.5	0–10	2.6	36.5
Symptom-specific well-being	6.6 (3.1)	7.5	0–10	28.8	8.1
QOL	5.0 (2.4)	5.0	0–10	6.1	5.9
Social Disability	1.1 (2.3)	0.0	0–10	3.3	72.5
Work Disability	0.8 (2.0)	0.0	0–10	2.6	82.9
Summary Score	3.7 (1.8)	3.6	0–8.8	0.0	2.9
EQ-5D					
Summary Score	0.8 (0.2)	0.8	−0.111–1.000	0.3	34.7
VAS	66.8 (22.8)	70.0	0–100	0.1	2.5
RDQ^a^	4.0 (5.5)	2.0	0–24	0.8	35.5
SF-8					
PCS	45.1 (8.7)	46.1	11.97–63.53	0.0	0.0
MCS	46.0 (8.6)	47.5	11.53–65.09	0.0	0.0
STarT^a^	2.7 (2.4)	2.0	0–9	2.7	21.9
Total					
Sub score (psychological)	1.5 (1.5)	1.0	0–5	5.5	35.5

COMI: Core Outcome Measures Index; SD: standard deviation; QOL: quality of life; EQ-5D: the EuroQol 5 Dimension; VAS: visual analogue scale; RDQ: Roland Morris Disability Questionnaire; SF-8: Short Form 8™ Health Survey; PCS: physical component summary; MCS: mental component summary; STarT: the Keele STarT Back Screening Tool

^a^Higher score indicates worse status; lower score indicates better status

#### Validity and internal consistency

Convergent validity was evaluated by assessing the correlations between the Japanese COMI scores and the scores on the relevant reference questionnaires that measure the same or similar constructs. All the COMI domain scores and the COMI summary score correlated significantly with the respective reference questionnaires [Table 4]. Correlation coefficients met the expectation of indicating strong correlations (≥0.5) for all except for symptom-specific well-being (− 0.48 and − 0.33 with SF-8 physical component summary and mental component summary, respectively) and the disability domains (0.48 with STarT total), which indicated just moderate correlations. Correlations between the COMI summary scores and all the reference questionnaires were the strongest (− 0.52 to − 0.72).

**Table 4:** Correlations^a^ between the COMI and the related questionnaires and domains

	COMI					
	Pain	Function	Symptom-specific well-being	QOL	Disability	Summary
EQ-5D Summary index	−0.60	−0.58	−0.54	− 0.60	− 0.48	− 0.72
RDQ	0.64	0.63	0.52	0.52	0.54	0.71
SF-8 PCS	−0.55	−0.62	−0.48	− 0.50	−0.43	− 0.66
SF-8 MCS	−0.36	− 0.46	−0.33	− 0.54	−0.34	− 0.52
STarT Total	0.63	0.60	0.53	0.57	0.48	0.71
STarT Sub score (psychological)	0.56	0.56	0.50	0.55	0.43	0.67

All the correlations were significant (*P* < 0.001)

^a^Spearman Rank correlation coefficients

COMI: Core Outcome Measures Index; QOL: quality of life; EQ-5D: the EuroQol 5 Dimension; RDQ: Roland Morris Disability Questionnaire; SF-8: Short Form 8™ Health Survey; PCS: physical component summary; MCS: mental component summary; STarT: the Keele STarT Back Screening Tool

**Fig. 2:**
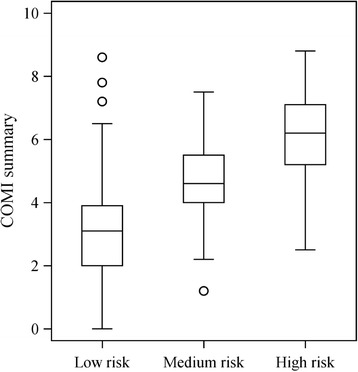
Box plots of the COMI summary score by prognostic risk groups(*a*). *a* Participants were stratified into three prognostic risk groups based on the total score of the 9 questions and on the sub score for the 5 psychological questions of the STarT: low risk (total score of 0–3), medium risk (total score of ≥4 with sub score of ≤3), and high risk (sub score of ≥4) groups [21]. COMI: core outcome measures index; STarT: the Keele STarT Back Screening Tool

The known-group validity was evaluated by comparing the COMI summary score among the low, middle, and high risk groups as measured with the STarT. The median COMI summary score was higher in the groups with higher prognostic risk (median [25th–75th percentile]: 3.1 [2.0–3.9], 4.6 [4.0–5.5], and 6.2 [5.2–7.1] in low, middle, and high prognostic risk groups, respectively) and demonstrated a significant, positive linear relationship with the prognostic risk level [Fig. 2] (*P* < 0.001, Jonckheere-Terpstra test).

Cronbach’s alpha for the Japanese version of the COMI was 0.82.

## Discussion

This study aimed to develop a Japanese version of the COMI. The cross-cultural adaptation process generated a Japanese version of the COMI that retained conceptual equivalence to the original, using comprehensible and acceptable Japanese expressions. Using a very large sample obtained from the general Japanese population, analyses of the psychometric properties of the Japanese COMI substantiated its validity.

We translated and linguistically validated the Japanese COMI based on published guidelines [13, 14], which facilitate a cross-cultural adaptation that retains equivalence to the original version. In the pilot test, all patients answered the Japanese COMI without any major problems regarding understanding or acceptability of the instrument. This suggests that the content of the Japanese COMI is equivalent to that of the original English version and uses expressions that are acceptable for Japanese patients.

Particularly high percentages for ceiling effects were observed for the social and work disability items. Other language versions [4, 5, 8–10] have also reported high percentages for ceiling effects for the disability domain or its items, although in none of these studies were the effects as pronounced as in the present study. The high percentages for ceiling effects that we documented may reflect the relatively low severity of LBP of the volunteers involved in the present study compared with those in previous studies: the proportion of individuals with no sick leave was 40% and 32.9% in Italian [10] and French [9] studies, respectively; and the mean (SD) RDQ scores were 13.5 (5.6), 11.7 (5.7), 10.5 (6.3), and 11.6 (5.1) in German [4], Brazilian-Portuguese [8], Italian [10], and French [9] studies, respectively. In the present study, recruitment did not take place in hospitals or clinics, but was done online and included more individuals with less severe LBP that did not require treatment or sickness leave. The potential consequences of high floor and ceiling effects are that they can render an instrument unresponsive, since transitions to even more extreme statuses are not measurable. However, both German [4] and Spanish versions [5] of the COMI have been shown to be responsive (effect size: 0.95 (for the response 6 months after surgical or conservative treatment) in German [4] and 1.04 (for the response after surgery) in Spanish versions [5]), despite relatively large floor/ceiling effects of > 15% (floor effects for symptom-specific well-being (49.6%) and disability (18.5%) in the German version [4], and for back function (40.3%), symptom-specific well-being (64.9%), and disability (38.3%) in the Spanish version [5]; and ceiling effects for back function (31.2%) and disability (29.8%) in the German version [4]). Hence, the influence of large ceiling effects on the responsiveness of the Japanese COMI summary score might also be expected to be limited.

In the assessment of convergent validity, consistent with previous validation studies [4, 5, 8–11] the Japanese COMI correlated strongly with the relevant reference questionnaires that measure the same or similar constructs. The disability domain correlated strongly with RDQ, which specifically reflects physical disability, but less strongly with STarT total. This corroborates the convergent validity of the COMI disability domain as a measure specifically targeting physical disability and correlating only moderately with STarT, which incorporates both physical disability and psychological factors. Despite the only moderate correlation with the COMI disability domain, the STarT total score (i.e., covering both physical and psychological aspects) correlated strongly with the COMI summary score, which reflects the influence of the back problem on many domains, substantiating the multidimensionality of the COMI. Finally, the scores for symptom-specific well-being correlated only moderately with the SF-8 physical component summary (PCS) and mental component summary (MCS) scores. Other language versions also reported relatively weak correlations between symptom-specific well-being and quality-of-life reference scales [5, 9, 10]. It was considered that this item may measure a particularly unique concept that differs from “quality of life”.

A previous study reported a linear increase in the number of LBP-related absences across the STarT risk groups [29]. Assuming that the number of absences reflects the impact of the back problem, in the same way that the multidimensional COMI score does, we also expected the COMI score to differ across the STarT prognostic risk levels. We hence evaluated known-group validity by examining the COMI scores for each of the prognostic risk levels. The result demonstrated a clear trend for a risk-associated increase in the COMI summary score. The trend suggests that the Japanese COMI is sensitive enough to reflect the level of prognostic risk.

For the purposes of comparing with other language versions, we calculated Cronbach’s alpha (internal consistency) for the Japanese version of the COMI. With a value of 0.82 it was similar to the values (0.75–0.92) reported for other language versions [4, 5, 11]. However, given that the COMI was originally designed as a multidimensional index (rather than a unidimensional scale), it is not actually considered necessary or even appropriate to determine its internal consistency [27].

There are some limitations to be considered when interpreting our findings. First, the number of subjects included in the pilot test of the pre-final version of the Japanese COMI may be considered small. However, 5–8 patients can probably be considered sufficient for pilot testing to assess issues and concerns regarding comprehensibility and conceptual equivalence, based on the recommendations of International Society for Pharmacoeconomics and Outcomes Research (ISPOR) to conduct cognitive debriefing in 5–8 persons [30]. Further, in usability studies it has been shown that testing in 5 persons gives you a grasp of problems, whereas much new is not observed after the 5th person [31–33]. We had intended to add more patients for further interview if major problems arose in the small group, which seemed not to be the case for this study. Second, the generalizability of the present results may be limited. Due to the nature of the online recruitment, some groups of individuals may be under-represented (e.g., those without access to the Internet) and others over-represented (e.g., those with a greater motivation to participate). Moreover, the present validation study limited participation to individuals aged 20–69 years with chronic LBP (≥3 months). Third, the study did not validate the “worst problem” item from the Spine Tango patient-assessment form, incorporated into our questionnaire battery. There may remain a need for future validation of the Japanese version of this single item, which is independent of the COMI summary score calculation. Finally, this study did not evaluate the test-retest reliability of the Japanese COMI. We first wanted to ensure that the Japanese COMI would measure what it was intended to measure, i.e. showed construct validity, to consolidate the ground for future examinations of its consistency and test-retest reliability. Further assessments of reliability are warranted prior to the use of the Japanese COMI in actual clinical or research settings.

## Conclusions

We developed a Japanese version of the COMI that displayed qualities that support its convergent and known-group validity. The Japanese COMI represents a practical tool to capture the multidimensional impact of chronic LBP in Japanese patients. The availability of a Japanese version should facilitate the widespread use of the COMI and promote the standardization and accumulation of data, allowing improved documentation of the care received by patients with chronic LBP.

## Additional file

Additional file 1: Japanese version of the Core Outcome Measures Index. Japanese version of the Core Outcome Measures Index in Japanese. (PDF 232 kb)

## Abbreviations

COMI: Core Outcome Measures Index
EQ-5D: EuroQol 5 Dimension
LBP: Low back pain
MCS: Mental component summary
PCS: Physical component summary
RDQ: Roland Morris Disability Questionnaire
STarT: Keele STarT Back Screening Tool

## References

[1] Pengel LH, Herbert RD, Maher CG, Refshauge KM (2003). Acute low back pain: systematic review of its prognosis. BMJ.

[2] van Tulder M, Becker A, Bekkering T, Breen A, del Real MT, Hutchinson A, Koes B, Laerum E, Malmivaara A (2006). COST B13 working group on guidelines for the Management of Acute low Back Pain in primary care. Chapter 3. European guidelines for the management of acute nonspecific low back pain in primary care. Eur Spine J.

[3] Deyo RA, Battie M, Beurskens AJ, Bombardier C, Croft P, Koes B, Malmivaara A, Roland M, Von Korff M, Waddell G. Outcome measures for low back pain research. A proposal for standardized use. Spine (Phila Pa 1976) 1998;15. 23:2003–13. 10.1097/00007632-199809150-00018 9779535

[4] Mannion AF, Elfering A, Staerkle R, Junge A, Grob D, Semmer NK, Jacobshagen N, Dvorak J, Boos N (2005). Outcome assessment in low back pain: how low can you go?. Eur Spine J.

[5] Ferrer M, Pellisé F, Escudero O, Alvarez L, Pont A, Alonso J, Deyo R (2006). Validation of a minimum outcome core set in the evaluation of patients with back pain. Spine (Phila Pa 1976).

[6] Mannion AF, Porchet F, Kleinstück FS, Lattig F, Jeszenszky D, Bartanusz V, Dvorak J, Grob D (2009). The quality of spine surgery from the patient's perspective. Part 1: the Core outcome measures index in clinical practice. Eur Spine J.

[7] White P, Lewith G, Prescott P. The core outcomes for neck pain: validation of a new outcome measure. Spine (Phila Pa 1976) 2004;1. 29:1923–30. 10.1097/01.brs.0000137066.50291.da 15534418

[8] Damasceno LH, Rocha PA, Barbosa ES, Barros CA, Canto FT, Defino HL, Mannion AF (2012). Cross-cultural adaptation and assessment of the reliability and validity of the Core outcome measures index (COMI) for the Brazilian-Portuguese language. Eur Spine J.

[9] Genevay S, Cedraschi C, Marty M, Rozenberg S, De Goumoëns P, Faundez A, Balagué F, Porchet F, Mannion AF (2012). Reliability and validity of the cross-culturally adapted French version of the Core outcome measures index (COMI) in patients with low back pain. Eur Spine J.

[10] Mannion AF, Boneschi M, Teli M, Luca A, Zaina F, Negrini S, Schulz PJ (2012). Reliability and validity of the cross-culturally adapted Italian version of the Core outcome measures index. Eur Spine J.

[11] Nakhostin Ansari N, Naghdi S, Eskandari Z, Salsabili N, Kordi R, Hasson S (2016). Reliability and validity of the Persian adaptation of the Core outcome measure index in patients with chronic low back pain. J Orthop Sci.

[12] Fankhauser CD, Mutter U, Aghayev E, Mannion AF (2012). Validity and responsiveness of the Core outcome measures index (COMI) for the neck. Eur Spine J.

[13] Beaton DE, Bombardier C, Guillemin F, Ferraz MB. Guidelines for the process of cross-cultural adaptation of self-report measures. Spine (Phila Pa 1976) 2000;15. 25:3186–91. 10.1097/00007632-200012150-00014 11124735

[14] Guillemin F, Bombardier C, Beaton D (1993). Cross-cultural adaptation of health-related quality of life measures: literature review and proposed guidelines. J Clin Epidemiol.

[15] Pochon L, Kleinstück FS, Porchet F, Mannion AF (2016). Influence of gender on patient-oriented outcomes in spine surgery. Eur Spine J.

[16] Mannion AF, Mutter UM, Fekete TF, Porchet F, Jeszenszky D, Kleinstück FS (2014). Validity of a single-item measure to assess leg or back pain as the predominant symptom in patients with degenerative disorders of the lumbar spine. Eur Spine J.

[17] Spine Tango forms. http://www.eurospine.org/forms.htm. Accessed at November 8, 2016.

[18] Von Korff M, Ormel J, Keefe FJ, Dworkin SF (1992). Grading the severity of chronic pain. Pain.

[19] Rabin R, Charro FD (2001). EQ-SD: a measure of health status from the EuroQol group. Ann Med.

[20] Roland M, Morris RA (1983). Study of the natural history of back pain. Part I: development of a reliable and sensitive measure of disability in low-back pain. Spine (Phila Pa 1976).

[21] Fukuhara S, Suzukamo Y. Manual of the SF-8 Japanese version, (in Japanese). Kyoto: Institute for Health Outcomes and Process Evaluation Research, 2004.

[22] Hill JC, Dunn KM, Lewis M, Mullis R, Main CJ, Foster NE, Hay EM (2008). A primary care back pain screening tool: identifying patient subgroups for initial treatment. Arthritis Rheum.

[23] McHorney CA, Tarlov AR (1995). Individual-patient monitoring in clinical practice: are available health status surveys adequate?. Qual Life Res.

[24] Cohen J (1988). Statistical power analysis for the behavioral sciences.

[25] Jonckheere ARA (1954). Distribution-free k-sample test against ordered alternatives. Biometrika.

[26] Terpstra TJ (1952). The asymptotic normality and consistency of Kendall's test against trend, when ties are present in one ranking. Indag Math.

[27] Streiner DL, Norman GR, Cairney J (2015). Health measurement scales: a practical guide to their development and use.

[28] Nunnally JC (1978). Psychometric theory.

[29] Matsudaira K, Oka H, Kikuchi N, Haga Y, Sawada T, Tanaka S (2016). Psychometric properties of the Japanese version of the STarT back tool in patients with low back pain. PLoS One.

[30] Wild D, Grove A, Martin M, Eremenco S, McElroy S, Verjee-Lorenz A (2005). ISPOR task force for translation and cultural adaptation. Principles of good practice for the translation and cultural adaptation process for patient-reported outcomes (PRO) measures: report of the ISPOR task force for translation and cultural adaptation. Value Health.

[31] Jakob N, Landauer TK. A mathematical model of the finding of usability problems. Proceedings of the INTERACT'93 and CHI'93 conference on human factors in computing systems. ACM. 1993:206–13.

[32] Nielsen Norman Group. Why You Only Need to Test with 5 Users. Nielsen Norman Group. https://www.nngroup.com/articles/why-you-only-need-to-test-with-5-users/ Accessed 13 September 2017.

[33] Nielsen Norman Group. Heow Many Test Users in a Usability Study? Nielsen Norman Group. https://www.nngroup.com/articles/how-many-test-users/ Accessed 13 September 2017.

[34] Ikeda S, Ikegami N (2002). Preference-based measure (focus on EQ-5D).

