# Supplementary material for: Development of the Japanese Core Outcome Measures Index (COMI): cross-cultural adaptation and psychometric validation
Source: BMC Musculoskelet Disord. 2018 Mar 2;19:71. doi: 10.1186/s12891-018-1986-x (PMC5834844; doi:10.1186/s12891-018-1986-x)
Supplement: Supplementary file 1 — Japanese version of the Core Outcome Measures Index. Japanese version of the Core Outcome Measures Index in Japanese. (PDF 232 kb) [file 12891_2018_1986_MOESM1_ESM.pdf]

腰や背中の不調で治療を受けている方は、 **A** の質問にすべてお答えください（黄色用紙）。

頚（くび）の不調で治療を受けている方は、 **B** の質問にすべてお答えください（青色用紙）。

A

腰や背中の不調は、腰や背中の痛み、かつ/または脚（あし）/でん部（おしり）の痛み、または、ピリピリ感、チクチク感、しびれ、といった感覚の障害につながることがあります。

1 以下のうち**最も**困るのはどの不調ですか。**1つだけ**選んでチェック（✔）をつけてください。

- ☐1 腰や背中の痛み
- ☐2 脚（あし）/でん部（おしり）の痛み
- ☐3 腰や背中、脚（あし）、でん部（おしり）の感覚の障害（例. ピリピリ感、チクチク感、しびれなど）
- ☐4 上記に該当なし

2 以下の２問（2a と 2b）について、あなたの痛みの強さを回答してください。
回答は、0 から 10 までの線上に×印をつけてください。
0 は**痛みなし**、10 はあなたの**想像できる最もひどい痛み**です。
質問は、**腰や背中の痛み**と、**脚（あし）の痛み（坐骨神経痛）/でん部（おしり）の痛み**の２つにわかれています。

例：
痛みなし　　　　想像できる最もひどい痛み
0　1　2　3　4（×）　5　6　7　8　9　10

2a 先週、**腰や背中の痛み**の強さはどの程度でしたか。

痛みなし　　　　想像できる**最もひどい**痛み
0　1　2　3　4　5　6　7　8　9　10

2b 先週、**脚（あし）の痛み（坐骨神経痛）/でん部（おしり）の痛み**の強さはどの程度でしたか。

痛みなし　　　　想像できる**最もひどい**痛み
0　1　2　3　4　5　6　7　8　9　10

3 ここ１週間、あなたの腰や背中の不調で、普段の仕事（**仕事と家事の両者を含む**）**がどの程度妨げられましたか。**

- ☐1 まったく妨げられなかった
- ☐2 わずかに妨げられた
- ☐3 まあまあ妨げられた
- ☐4 かなり妨げられた
- ☐5 非常に妨げられた

4 **現在の症状が今後一生続く**としたら、どう感じますか。

- ☐1 とても満足
- ☐2 やや満足
- ☐3 満足でも不満でもない
- ☐4 やや不満
- ☐5 とても不満

次のページもお答えください

A

5 過去７日間をふり返ってください。あなたの生活の質（QOL）を、どのように評価しますか。

- □1 とても良い
- □2 良い
- □3 良くも悪くもない
- □4 悪い
- □5 とても悪い

6 ここ４週間、あなたの腰や背中の不調のために、普段していること（仕事、家事、学業、趣味の活動や娯楽）を何日減らしましたか。

- □1 減らしていない
- □2 1～7日
- □3 8～14日
- □4 15～21日
- □5 22日以上

7 ここ４週間、あなたの腰や背中の不調で、仕事（仕事、学校、家事）に行くことができない日が何日ありましたか。

- □1 なかった
- □2 1～7日
- □3 8～14日
- □4 15～21日
- □5 22日以上

日付：＿＿＿＿＿＿＿＿＿＿ 署名：＿＿＿＿＿＿＿＿＿＿

お答えいただき、心より感謝します

腰や背中の不調は、腰や背中の痛み、かつ/または脚/でん部（おしり）の痛み、または、ピリピリ感、チクチク感、しびれ、といった感覚の障害につながることがあります。

1 以下のうち<u>最も</u>困るのはどの不調ですか。<u>1つだけ</u>選んでチェック（✔）をつけてください。

- □1 腰や背中の痛み
- □2 脚/でん部（おしり）の痛み
- □3 腰や背中、脚、でん部（おしり）の感覚の障害（例．ピリピリ感、チクチク感、しびれなど）
- □4 上記に該当なし

2 以下の２問（2a と 2b）について、あなたの痛みの強さを回答してください。
回答は、0 から 10 までの線上に×印をつけてください。
0 は**痛みなし**、10 はあなたの**想像できる最もひどい痛み**です。
質問は、**腰や背中の痛み**と、**脚の痛み（坐骨神経痛）/でん部（おしり）の痛み**の２つにわかれています。

例：
痛みなし ………………… 想像できる最もひどい痛み
0 1 2 3 4 5 6 7 8 9 10 （4 に×印）

2a 先週、**腰や背中の痛み**の強さはどの程度でしたか。

痛みなし ………………… 想像できる**最もひどい**痛み
0 1 2 3 4 5 6 7 8 9 10

2b 先週、**脚の痛み（坐骨神経痛）/でん部（おしり）の痛み**の強さはどの程度でしたか。

痛みなし ………………… 想像できる**最もひどい**痛み
0 1 2 3 4 5 6 7 8 9 10

3 ここ１週間、あなたの腰や背中の不調で、**普段の仕事（仕事と家事の両者を含む）がどの程度妨げられましたか。**

- □1 まったく妨げられなかった
- □2 わずかに妨げられた
- □3 まあまあ妨げられた
- □4 かなり妨げられた
- □5 非常に妨げられた

4 **現在の症状が今後一生続く**としたら、どう感じますか。

- □1 とても満足
- □2 やや満足
- □3 満足でも不満でもない
- □4 やや不満
- □5 とても不満

5 **過去７日間**をふり返ってください。あなたの生活の質（QOL）を、どのように評価しますか。

- □1 とても良い
- □2 良い
- □3 良くも悪くもない
- □4 悪い
- □5 とても悪い

次のページもお答えください

6 **ここ４週間**、あなたの腰や背中の不調のために、**普段していること**（仕事、家事、学業、趣味の活動や娯楽）**を何日減らしましたか。**

- □$_1$ 減らしていない
- □$_2$ 1～7日
- □$_3$ 8～14日
- □$_4$ 15～21日
- □$_5$ 22日以上

7 **ここ４週間**、あなたの腰や背中の不調で、仕事（仕事、学校、家事）に**行くことができない**日が何日ありましたか。

- □$_1$ なかった
- □$_2$ 1～7日
- □$_3$ 8～14日
- □$_4$ 15～21日
- □$_5$ 22日以上

8a 1年前の手術の結果、何か**合併症**が起きましたか（例．創（きず）の治りの問題、麻痺、感覚の障害）。

- □$_1$ いいえ
- □$_2$ はい → 合併症を記載してください：______________________________

8b 合併症で、どの程度悩まされましたか？

- □$_1$ まったく悩まされていない
- □$_2$ わずかに悩まされた
- □$_3$ まあまあ悩まされた
- □$_4$ かなり悩まされた
- □$_5$ 非常に悩まされた

9 **1年前の手術から**、何らかの腰椎（腰や背中）の**追加**手術を受けましたか。

- □$_1$ いいえ
- □$_2$ はい、しかし脊椎のレベル（背骨の場所）が異なります
- □$_3$ はい、同じ脊椎のレベル（同じ背骨の場所）です

10 あなたの腰や背中の不調に対する**治療を通し、当院での**医療（ケア）全般に、どの程度満足しましたか。

- □$_1$ とても満足
- □$_2$ やや満足
- □$_3$ 満足でも不満でもない
- □$_4$ やや不満
- □$_5$ とても不満

11 全体的にみて、**1年前の手術は**、あなたの腰や背中の不調を、どの程度改善しましたか。

- □$_1$ とても改善した
- □$_2$ 改善した
- □$_3$ 少ししか改善しなかった
- □$_4$ 改善しなかった
- □$_5$ 悪化した

日付：______________________　署名：__________________

お答えいただき、心より感謝します
